# Aseptic Encephalitis in an Immunocompetent Young Adult: A Case Report

**DOI:** 10.7759/cureus.89821

**Published:** 2025-08-11

**Authors:** Abigail J Saldaña Solano, Karina Carrillo Loza, Juan Benítez Valenzuela, Edgar Rangel López

**Affiliations:** 1 Cell Reprogramming Laboratory, Instituto Nacional de Neurología y Neurocirugía Manuel Velasco Suárez, Mexico City, MEX; 2 Neurology, Instituto Nacional de Neurología y Neurocirugía Manuel Velasco Suárez, Mexico City, MEX

**Keywords:** aseptic encephalitis, encephalitis of unknown etiology, neurotropic viruses, oligoclonal bands, viral encephalitis

## Abstract

Encephalitis is a major diagnostic challenge, especially when clinical findings, imaging studies, and cerebrospinal fluid (CSF) analysis suggest parenchymal encephalitis, but no specific etiologic agent or detectable autoimmunity can be identified.

We present the case of a 27-year-old man with subacute neuropsychiatric symptoms following an infectious prodromal stage. Neuroimaging studies revealed frontotemporal involvement, and oligoclonal bands (OCBs) were detected in the CSF, without suggesting an infectious or autoimmune etiology.

The monophasic clinical course, the absence of relapses, the negative antibody test, and the clinical stability over time supported the diagnosis of aseptic encephalitis. This case report discusses the magnetic resonance imaging findings, highlighting the role of OCBs as a nonspecific marker of immune activation, and the utility of the 2016 Graus diagnostic criteria to rule out probable seronegative autoimmune encephalitis.

This case underscores the importance of interpreting immunological findings within a clinical context and applying established diagnostic criteria for different types of encephalitis to prevent misdiagnosis and minimize the risks associated with unnecessary immunosuppressive treatment.

## Introduction

Encephalitis is a neurological syndrome with an acute or subacute course, characterized by inflammation of the brain parenchyma and clinically manifested by altered mental status, fever, seizures, and focal neurological deficits [1]. In approximately 50% of cases, encephalitis is secondary to an infectious process [2], most commonly caused by neurotropic viruses [3]. However, among these infection-related cases, no specific etiologic agent can be identified in nearly half [4], which leads to the diagnosis of aseptic encephalitis.

Aseptic encephalitis is defined as an inflammatory condition of the central nervous system (CNS) with clinical, imaging, or cerebrospinal fluid (CSF) findings consistent with encephalitis, but without isolation of a clear infectious agent or evidence of demonstrable autoimmunity.

This report presents the case of a young adult patient with a history of acute infectious disease, a neuropsychiatric course, and neuroimaging findings suggestive of predominantly frontotemporal encephalitis. In this case, neither an infectious agent nor evidence of autoimmunity was found. This case illustrates the diagnostic difficulties of aseptic encephalitis and provides an opportunity to reflect on possible endemic neurotropic viral pathogens that are no longer detectable in late stages.

## Case presentation

A 27-year-old immunocompetent male patient, who was previously healthy, had a normal level of development, with no significant past medical history. He had denied smoking, alcoholism, or drug abuse. There was no family history of neurological, psychiatric, or autoimmune diseases. The patient was enrolled in university studies with good academic performance until the onset of symptoms and had no significant occupational exposures. In April 2015, he presented with a severe headache after a dental procedure, followed by symptoms of general malaise and drowsiness. A week later, he exhibited behavioral changes, including disinhibition, emotional lability, and impulsive behavior. He was evaluated in a private hospital where a lumbar puncture (LP) and magnetic resonance imaging (MRI) were reportedly performed, which did not reveal any abnormalities. He was diagnosed with presumed viral encephalitis and treated empirically with intravenous acyclovir (10 mg/kg every eight hours for 14 days), showing partial clinical improvement.

**Figure 1:**
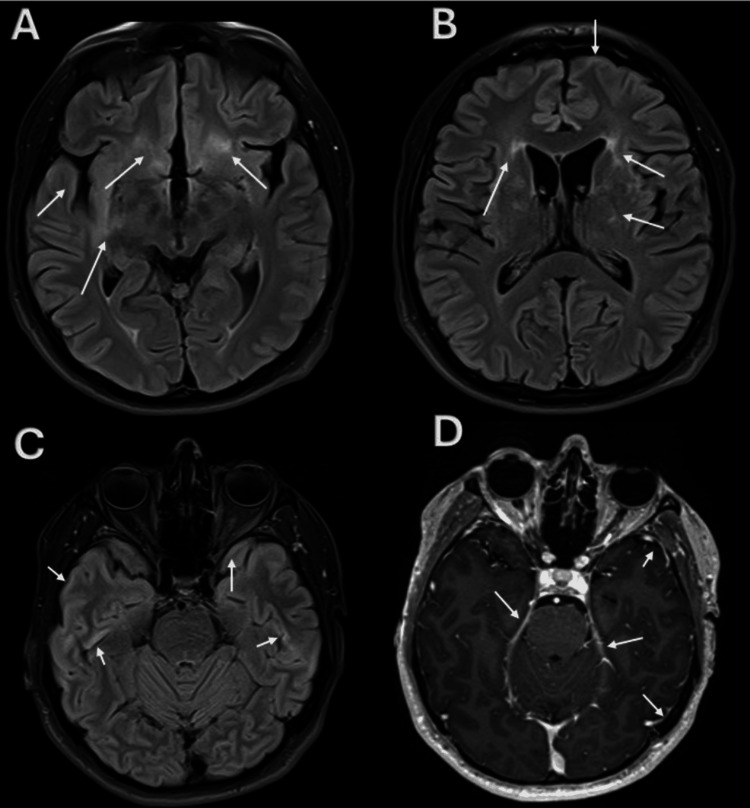
Axial magnetic resonance imaging (MRI). (A, B) Fluid-attenuated inversion recovery (FLAIR) sequence shows reduced brain volume in the temporal region and hyperintense areas in the bilateral frontotemporal region, primarily around the anterior horns of the lateral ventricles. (C) Cortical hyperintensity in the temporal lobes. (D) Axial post-contrast section showing leptomeningeal enhancement in the bilateral temporal cortex. Arrows indicate areas of pathological signal. Images correspond to the patient's initial MRI and are published with informed consent. All identifying information has been removed.

In June 2016, he was referred to our hospital due to residual symptoms. Neurological examination revealed emotional lability, preserved cranial nerve function, and mild right-side motor asymmetry (proximal lower limb strength 4/5). Cognitive testing revealed mild impairments in abstraction and calculation (Montreal Cognitive Assessment (MoCA) score: 23/30). The following investigations were performed: (1) MRI of the brain showed findings consistent with encephalitis with right insular involvement described as hyperintensity in the periventricular white matter in the frontal recesses, corona radiata/lateral and basal ganglia (Figure 1). (2) Normal CSF analysis. (3) Negative results for anti-N-methyl-D-aspartate receptor antibodies (anti-NMDAr). (4) Negative CSF film array by polymerase chain reaction (PCR). (5) Non-reactive serology for human immunodeficiency virus antibodies (anti-HIV), hepatitis B virus antibodies (anti-HBV), and hepatitis C virus antibodies (anti-HCV), and a negative Venereal Disease Research Laboratory (VDRL) test. (6) Negative rheumatologic screening profile. (7) Presence of oligoclonal bands (OCBs) in the CSF.

The presence of OCBs in the CSF was considered a non-specific finding that did not correlate clinically or radiologically with a demyelinating disease.

Based on the monophasic course preceded by constitutional symptoms, the neurotropic pattern of damage predominantly in the orbitofrontal and temporal cortex, the presence of leptomeningeal enhancement, and the absence of relapses over a 10-year follow-up period, a final diagnosis of aseptic encephalitis of probable viral etiology was made.

The therapeutic plan, including pharmacological management with clozapine (initial dose: 12.5 mg daily, titrated to 100 mg daily) and paroxetine (20 mg daily) for behavioral and mood stabilization, as well as structured psychoeducation for family members, was thoroughly discussed and accepted by both the patient and his caregivers. Clinical improvement in behavioral symptoms was noted within four weeks of initiating therapy. The patient has demonstrated progressive and sustained clinical improvement. He remains under active follow-up by a multidisciplinary outpatient team, which includes neurology, psychiatry, and neuropsychology, and is currently undergoing further evaluation to optimize long-term management. No evidence of clinical relapse or functional decline has been observed to date.

## Discussion

Encephalitis represents a major diagnostic challenge, especially when the patient presents with clinical symptoms consistent with a viral cause of infection, but etiologic investigations are negative.

In the present case, the patient exhibited neuropsychiatric changes that developed over several days to a few weeks, consistent with a subacute onset. Neuroimaging findings showed frontotemporal involvement, and OCBs were present in the CSF, without identification of a specific etiologic agent or evidence of autoimmune encephalitis. These findings support the diagnosis of aseptic encephalitis, a recognized entity within the spectrum of central nervous system inflammatory syndromes, typically associated with a probable but unconfirmed infectious cause.

One of the most important contributions to the understanding of this entity comes from the California Encephalitis Project (1997-2010), a multicenter cohort that analyzed more than 5,000 cases of encephalitis in the United States. Despite the use of advanced diagnostic algorithms, no etiology could be identified in approximately 50% of cases, underscoring the frequency and clinical relevance of aseptic encephalitis. In addition, the study highlighted that some patients with a clinical profile suggestive of viral infection did not yield conclusive microbiologic evidence, prompting a reassessment of diagnostic windows and available methods for virus detection.

**Figure 2:**
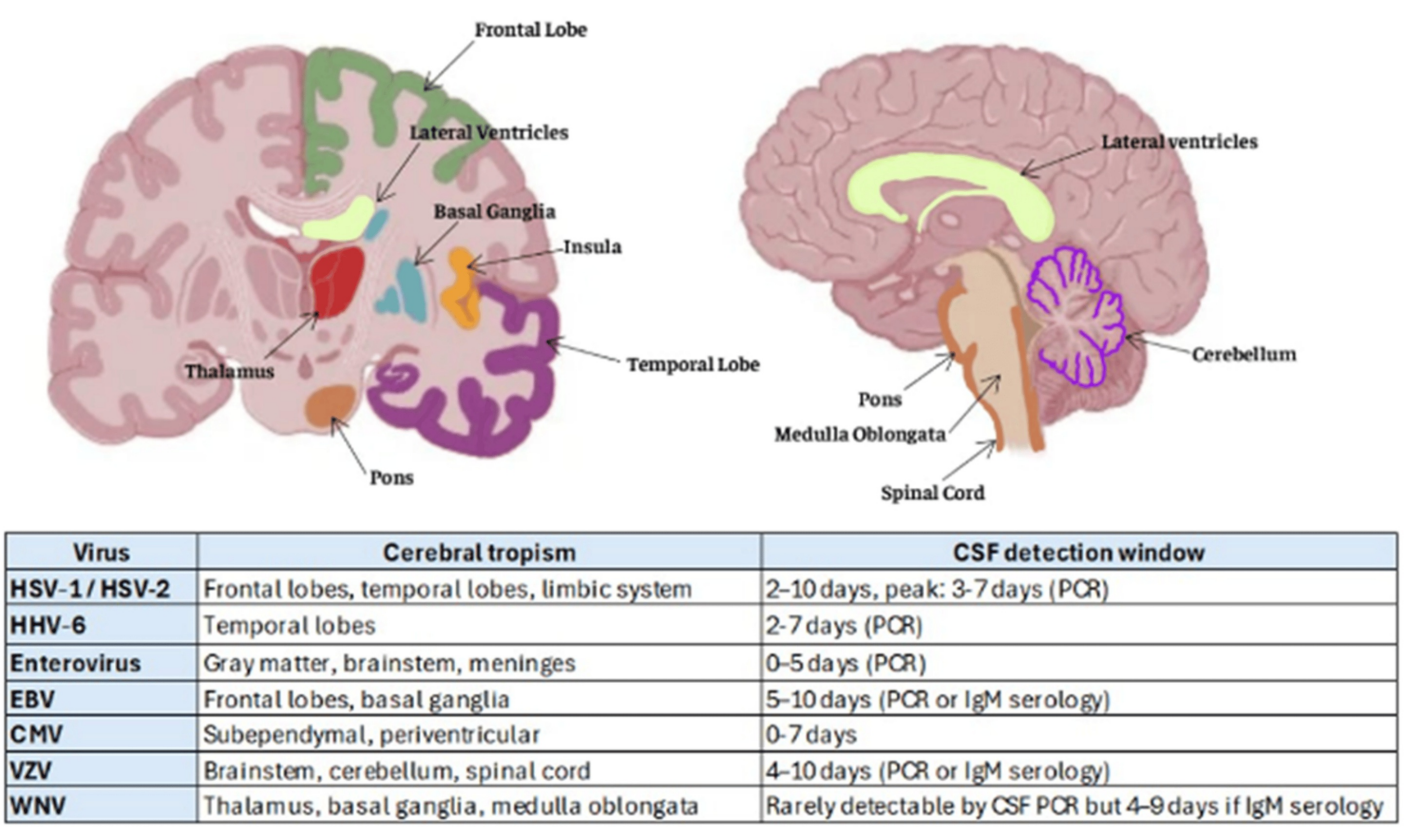
Common viruses associated with encephalopathies, brain tropisms, and diagnostic detection windows. Schematic representation of brain regions typically affected by common encephalitis-associated viruses, based on their neurotropism, and their corresponding cerebrospinal fluid (CSF) detection windows using polymerase chain reaction (PCR) or immunoglobulin M (IgM) serology. Highlighted brain areas in different colors indicate anatomical regions most frequently involved in viral infection. Figure created using https://BioRender.com. Based on data from Saraya et al. [5], Yao et al. [6], and Smuts and Lamb [7]. CMV, cytomegalovirus; EBV, Epstein-Barr virus; VZV, varicella-zoster virus; WNV, West Nile virus; HSV-1, herpes simplex virus-1; HHV-6, human herpesvirus-6

From a clinical and imaging perspective, this patient had involvement of the right insula and frontotemporal white matter. These regions are frequently affected by neurotropic viruses such as herpes simplex virus-1 (HSV-1) [5], enterovirus, or HHV-6 (Figure 2) [6,7]. Although these viruses have not been detected by CSF PCR or serology, the possibility of a rapidly resolving or low-replicating viral infection, in which the virus leaves an immunologic footprint without being detectable by conventional methods, should be considered in conjunction with the late time point at which the CSF film array was performed. 

The presence of OCBs in CSF is a non-specific finding reflecting B-cell activation and intrathecal immunoglobulin synthesis. OCB positivity occurs in numerous neurologic diseases, both infectious and autoimmune (Table 1), including herpesvirus encephalitis, neurosyphilis, neurosarcoidosis, lupus encephalopathy, and paraneoplastic diseases. Even after the clinical symptoms have resolved, OCBs can persist for months to years due to a residual immune response or non-specific stimulation of the central nervous system [8]. For this reason, the need to interpret the presence of OCBs in the general clinical context is emphasized in order to avoid overdiagnosis of pathologies such as autoimmune diseases and to adequately consider resolved infectious etiologies that may cause persistent immune responses.

**Table 1:** Patterns of oligoclonal bands (OCBs) in cerebrospinal fluid (CSF) and serum, and their clinical implications. The table was created by the authors based on data from Jin et al. [8]. CNS, central nervous system

Typical OCB patterns for CSF	Presence in CSF/serum	Cause/indicator
OCBs (Type 1)	Negative/normal: absence of OCBs in CSF and serum	It usually indicates a normal immune response in the CNS.
OCBs (Type 2)	CSF positive/specific: OCBs in CSF but not in serum	Indicates intrathecal synthetic immunoglobulin, due to a limited and persistent response of B lymphocyte clones
OCBs (Type 3)	Positive/more detectable in CSF: multiple bands appear in both CSF and serum, but it is more specific in serum.	An immune response against specific infectious agents or an immune process that activates certain types of B lymphocytes to produce antibodies.
OCBs (Type 4)	Identical positive: identical OCBs in CSF and serum	Disruption of the hematoencephalic barrier (blood-brain barrier)
OCBs-Type 5	Monoclonal: identical monoclonal pattern.	Plasma cell disease instead of intrathecal immunoglobulin synthesis

In this strict sense, it is advisable to rely on criteria such as those described by Graus in 2016 to exclude seronegative autoimmune encephalitis from aseptic encephalitis. In the present case, the patient did not fulfill the main clinical criteria, nor did he show characteristic signs such as refractory epilepsy, dyskinesias, autonomic dysfunction, or a progressive neuropsychiatric syndrome [9]. The absence of specific antibodies in CSF and serum and clinical stability over time made it possible to exclude seronegative autoimmune encephalitis as the cause of the clinical picture. 

According to Dalmau and Graus [9], the diagnostic criteria for autoantibody-negative but probable autoimmune encephalitis are as follows:

(1) Rapid progression (<3 months) of working memory deficits (short-term memory loss), altered mental status, or psychiatric symptoms

(2) Exclusion of well-defined syndromes of autoimmune encephalitis: typical limbic encephalitis; Bickerstaff’s brainstem encephalitis; acute disseminated encephalomyelitis

(3) Absence of well-characterized autoantibodies in serum and CSF, and at least two of the following criteria: MRI abnormalities suggestive of autoimmune encephalitis; CSF pleocytosis, CSF-specific oligoclonal bands or elevated CSF IgG index, or both; brain biopsy showing inflammatory infiltrates and excluding other disorders (e.g., tumor)

(4) Reasonable exclusion of alternative causes: CNS infections (herpes simplex encephalitis, human herpesvirus-6 (HHV-6) encephalitis, neurosyphilis, Wgipple disease, HIV), metabolic or toxic encephalopathy (functional neurological disorders), drug toxicity (brain fog as a result of chemotherapy, polypharmacy)

These criteria have demonstrated utility for diagnosing autoimmune encephalitis, including antibody-negative disease, although clinicians should be mindful of potential pitfalls in atypical cases [9].

The correct evaluation of these criteria is clinically relevant, as a multicenter study by Flanagan and Geschwind in 2023 [10] reported that of 107 patients initially diagnosed with autoimmune encephalitis, 72% did not meet the Graus criteria, and 84 of them (≈79%) received immunotherapy without a true indication, with adverse effects such as psychosis, infection, or side effects of treatment occurring in 20 of cases. It was also found that around 27% of patients initially diagnosed with autoimmune encephalitis [10] had other diagnoses more related to functional or psychiatric disorders. This highlights the need to improve diagnostic accuracy to avoid unnecessary exposure to potentially risky immunosuppressive treatments.

## Conclusions

The case presented illustrates the difficulties in diagnosing encephalitis when a specific etiology cannot be determined. When findings are suggestive but inconclusive, further analysis of the case and a comprehensive clinical evaluation are required to avoid aggressive therapeutic decisions based solely on nonspecific biomarkers. The presence of OCBs in CSF should be interpreted with caution in the clinical context to avoid misdiagnosis of autoimmune diseases. Cases such as the one presented here highlight the need to continue developing more sensitive and specific diagnostic methods, as well as to further disseminate the information on aseptic encephalitis cases and establish specific criteria for this entity in order to achieve a more accurate and personalized approach in the future.
